# The effect of weight loss on brain age in schizophrenia spectrum disorders

**DOI:** 10.1371/journal.pmen.0000346

**Published:** 2025-09-02

**Authors:** Vittal Korann, Nicolette Stogios, Karen Marie Sandø Ambrosen, Gary Remington, Ariel Graff-Guerrero, Bjørn H. Ebdrup, Margaret Hahn, Sri Mahavir Agarwal

**Affiliations:** 1 Institute of Medical Science, University of Toronto, Toronto, Canada; 2 Centre for Addiction and Mental Health (CAMH), Toronto, Canada; 3 Center for Neuropsychiatric Schizophrenia Research and Center for Clinical Intervention and Neuropsychiatric Schizophrenia Research, Psychiatric Center Glostrup, Glostrup Hospital, University of Copenhagen, Glostrup, Denmark; 4 Department of Clinical Medicine, Faculty of Health and Medical Sciences, University of Copenhagen, Copenhagen, Denmark; 5 Department of Psychiatry, University of Toronto, Toronto, Canada; 6 Multimodal Imaging Group, Research Imaging Centre, Centre for Addiction and Mental Health (CAMH), Toronto, Canada; 7 Department of Pharmacology and Toxicology, University of Toronto, Toronto, Canada; 8 Banting and Best Diabetes Centre (BBDC), University of Toronto, Toronto, Canada; Beijing Normal University, CHINA

## Abstract

Individuals with schizophrenia spectrum disorders (SSDs) suffer from metabolic conditions including type 2 diabetes (T2D) and obesity. Moreover, they are at a high risk for cardiovascular disease and this could lead to a shortened life expectancy. Obesity is one of common comorbid conditions in SSDs, which has adverse effects on brain health. However, it is still unknown how metabolic disorders affect brain anatomy in SSDs, and the impacts of weight loss from pharmacological interventions are yet to be studied. This study includes a total of 48 patients with SSDs from three different clinical trials focusing on weight loss interventions. We acquired metabolic parameters, brain anatomical MRI, body mass index (BMI), cognition, and psychopathology scores at baseline and endpoint. We used a convolutional neural network-based classifier to calculate each patient’s brain-age gap estimate (brainAGE) based on high-quality brain structural T1 images. We examined the relationship between the reduction in BMI and brainAGE between two timepoints. There was a significant reduction in BMI (p < 0.001) between two timepoints. Additionally, the analysis revealed that none of the cognitive, or psychopathology measures demonstrated significant differences between the timepoints (p > 0.05). The results of the multiple regression analysis showed a positive association between the reduction in BMI and brainAGE (F(2,44) = 3.69, p = 0.03). Furthermore, there were no noteworthy associations observed between brainAGE and the aforementioned parameters (p > 0.05). This study revealed a positive correlation between brainAGE and significant weight loss in SSDs with comorbid obesity.

## Introduction

Schizophrenia spectrum disorders (SSDs) encompass schizophrenia, schizoaffective disorder, delusional disorder, schizotypal personality disorder, schizophreniform disorder, brief psychotic disorder, and psychosis associated with substance use or medical conditions. These are multifaceted and chronic mental health disorders characterized by profound disruptions in cognitive processes, perceptual experiences, emotional responsiveness, and social interactions [1]. These debilitating disorders are linked with high morbidity and mortality [2]. Nearly 50% of the patients are obese, and the chances of developing type 2 diabetes is 3–9 times higher than the healthy population [3]. Particularly, comorbid conditions like overweight and obesity are often observed in patients with SSDs [4,5] even in the initial stages of the disorder, including those experiencing the first episode of psychosis (FEP) [6]. Antipsychotic medications, which are necessary for the treatment of SSDs, alarmingly increase the risk of obesity [7]. A recent meta-analysis found that patients who were new to antipsychotics experienced a higher incidence of clinically significant weight gain than patients who were switching to other antipsychotics [8]. Furthermore, there is convincing proof to show that weight gain persists throughout antipsychotic treatment, with most of it occurring in the first six months [9]. Obesity is not only linked with worsening physical health and declined life expectancy [10], but it is also related to cognitive impairment in individuals with SSDs [11].

It has been shown that the brain is one of the end organs for impacts of obesity-linked damage in SSDs [12]. Studies have used the body mass index (BMI) as a surrogate indicator for measuring obesity’s adverse effects on brain health and cognition [13]. Research found that persons with obesity have more neurostructural abnormalities even without the presence of other diseases [14,15], and they tend to show higher brain ages compared to normal weight [16]. Such premature brain ageing may result from obesity-induced changes in the structural configuration of white and grey matter [17–19]. The deteriorations of grey matter are similar to what is seen in age-dependent patterns, suggesting that it is imperative to prevent obesity for normal brain ageing [18]. Furthermore, many studies found that the cardiometabolic complications associated with obesity including-hypertension, inflammation, dyslipidemia, and insulin resistance are linked to discernible anatomical changes in the brain [20–22]. Besides, risk factors such as dyslipidemia, high blood pressure, diabetes, markers of liver and kidney dysfunction, stroke history, high BMI, and smoking are also linked with premature brain ageing [23–25]. Therefore, these results highlight the importance of obesity prevention for normal brain ageing in patients with SSDs.

Recently, many studies have utilized medications including metformin, glucagon-like peptide-1 receptor agonists (GLP-1), and topiramate to treat antipsychotic-induced weight gain and latest meta-analyses confirm their efficacy and safety [26,27]. While it is still unknown whether improved metabolic health enhances overall brain health. The machine learning approaches with access to extensive neuroimaging datasets and recent developments in neuroimaging analyses have elevated normative models and thorough examinations of brain anatomy. By employing these models, the biological age of brain can be estimated with the use of structural magnetic resonance imaging (MRI) [28,29]. Single measure, i.e., brain age is an outcome of combination of complex and diverse neurostructural changes. The Brain Age Gap Estimate (brainAGE) is calculated by taking the difference between the predicted brain age and the individual’s chronological age [30].

This study includes brain imaging data from clinical trials that were conducted at two centers to treat metabolic dysfunction induced by antipsychotics using metformin [31], exenatide [32,33], and topiramate (NCT02808533). We investigated the relationship between brainAGE and BMI with the help of this unique longitudinal dataset of patients with SSDs. We hypothesized that the notable reduction in BMI will be positively associated with the brainAGE between baseline and endpoint. We also explored the correlation between changes in cognition, psychopathology, and brainAGE.

## Methods

This is a secondary analysis of data collected from three distinct double-blind randomized control trials studying exenatide (NCT01794429), metformin (NCT02167620), and topiramate (NCT02808533) for antipsychotic-induced metabolic dysfunction. The exenatide study was conducted in Copenhagen, Denmark, while the metformin and topiramate studies were conducted in Toronto, Canada. The studies were reviewed and approved by their respective institutional Research Ethics Board.

1. Participants

A total of 48 patients with SSDs across the three studies were included in this analysis (exenatide: N = 9, metformin: N = 11, topiramate: N = 12, placebo: N = 16). The metformin and topiramate investigations were conducted with a 16-week intervention, and the exenatide study was conducted with a 3-month (12-week) intervention. Inclusion/exclusion criteria of these investigations are listed in Table A in S1 Text. All patients provided written informed consent for participation in the studies. Data extracted from each study included: diabetes history, height, weight, BMI (BMI = weight (kg)/ height (meters)²), waist circumference, type of antipsychotic administered, fasting glucose, insulin, total cholesterol, low-density lipoprotein (LDL), high-density lipoprotein (HDL), triglycerides (TG), and HOMA-IR (HOMA-IR, homoeostatic model assessment for insulin resistance). The Positive and Negative Syndrome Scale (PANSS) was utilized to assess the level of severity of clinical symptoms [34] and corresponding Cronbach’s alpha calculated for its subscales is included in Table B in S1 Text. Additionally, cognitive function was assessed via composite score of the Brief Assessment for Cognition in Schizophrenia (BACS) [35]. The first MRI scan was completed for patients who met all inclusion criteria before taking the first dose of randomized intervention and the endpoint MRI scan was acquired after completing the whole study procedure.

2. MRI acquisition

For the metformin and topiramate studies, MRI images were acquired using Siemens Skyra 3.0 T at St. Michael’s Hospital, Toronto, Canada. The whole-brain anatomical T1 MPRAGE images were obtained using the following parameters: Slice thickness, 0.9 mm; TR/TE, 2300/3.55 ms; Flip angle, 8 degrees; FOV, 256; Matrix size, 256x256. The TAO [32,33,36] study MRI images were acquired using a 3.0 T, Philips Achieva, whole-body MRI scanner (Philips Healthcare, Best, the Netherlands). The whole-brain anatomical T1 MPRAGE images were obtained using the following parameters: Slice thickness, 0.8 mm; TR/TE, 10.006/4.59 ms; Flip angle, 8 degrees; FOV, 240; Matrix size, 320x320. Each patient was informed to remain still during the scan, and foam pads were used to minimize head motion. No structural brain abnormalities were found in any of the T1 scans.

3. BrainAGE calculation

Participants’ brain age was estimated by employing convolution neural network (CNN) based machine learning. This method accurately predicts the brain age of individuals across a broad spectrum of ages [37] and is robust against variations among different scanners. This CNN model was trained using more than 23,000 subjects from different scanners and varying age ranges of 18–95. We applied the T1 image-based ensemble approach to predict each patient’s brain age. The chronological age of each participant at the time of the baseline and endpoint MRI scans was subtracted from the estimated brain ages at the baseline and endpoint, respectively. This subtraction resulted in two unique differentials, labeled as the baseline brainAGE and the endpoint brainAGE. The following step involved the calculation of the ‘ΔbrainAGE’ represented by the difference between the endpoint and baseline brainAGE. This metric provides a time trajectory of brain aging over the specified interval by considering the discrepancy between the predicted ages of the brain at different timepoints.

4. Statistical analyses

For this present secondary analysis, we conducted a post hoc power analysis using G*Power (version 3.1.9.4). Only exenatide trial conducted a priori power calculation for its primary outcome [32]; the remaining two studies were pilot in nature and did not include formal power calculations. Linear regression model with two predictors, a total sample size of 48, α = 0.05, and power = 0.80, the estimated effect size (Cohen’s f²) is 0.17, corresponding to a medium-to-large effect. This indicates that this study sample size provides sufficient power to detect effects of this magnitude in the context of our analysis.

Statistical analyses were done using SPSS version 25. For metabolic and other clinical variables such as cognition/psychopathology scales, paired t-tests were used to assess the changes between baseline and endpoint measures. The difference between endpoint and baseline measures such as BMI, brainAGE, total cholesterol, LDL and HDL cholesterol, cognition/psychopathology scales, and HOMA-IR will be prefixed with ‘Δ’ to indicate the changes between the two timepoints. Using Spearman correlation, we examined the association between ΔbrainAGE and other demographic variables, such as sex, education, and duration of illness, to include them as covariates in multiple regression analyses. In addition, the same correlation was utilized to test the relationships of change in metabolic parameters and cognition/psychopathology scales with ΔbrainAGE. Lastly, multiple regression analysis was conducted to investigate the association between ΔbrainAGE and ΔBMI.

## Results

Table 1 presents the demographic and clinical features of the study sample. The notable differences were observed with respect to weight (p < 0.001), BMI (p < 0.001), HDL cholesterol (p = 0.009), and total cholesterol (p = 0.048) between endpoint and baseline. Apart from these, no other metabolic parameters, psychopathology, and cognitive measures showed significant differences between the two timepoints. Furthermore, no significant change in brainAGE scores was observed between two timepoints (p = 0.40). The details about demographic and clinical characteristics of the medication and placebo groups were given in Tables C and D in S1 Text, respectively.

**Table 1:** Characteristics of whole sample at baseline and endpoint.

Variable	Baseline	Endpoint	t-stats	p-value
Sex (F:M)	21:27	21:27	–	–
Age (years)	34.56 ± 9.25	–	–	–
Education (years)	12.85 ± 2.3	–	–	–
Diagnosis; n (%)		–	–	–
Schizophrenia	35(72.9)			
Schizoaffective	12(25.0)			
Psychosis	1(2.1)			
Ethnicity; n (%)		–	–	–
Caucasian	32(66.6)			
Asian	6(12.5)			
Black	6(12.5)			
Hispanic	3(6.3)			
Diabetes status; n (%)		–	–	–
Diabetes/ Prediabetes	19(39.6)			
Non-diabetes	29(60.4)			
Illness duration (years)	11.48 ± 8.68	–	–	–
CPZ equivalents	628.33 ± 1101.22	–	–	–
Weight^a^ (kg)	104.43 ± 22.10	102.15 ± 23.26	3.81	< 0.001
BMI^a^ (kg/m2)	35.94 ± 5.48	35.07 ± 5.87	4.22	< 0.001
Waist circumference (cm)	113.01 ± 15.14	110.70 ± 16.58	1.83	0.076
Fasting glucose (mmol/L)	5.57 ± 0.66	5.89 ± 1.76	-1.43	0.158
Insulin (pmol/L)	95.9 ± 63.65	107.07 ± 88.47	-0.60	0.55
HOMA IR index	4.21 ± 2.92	3.21 ± 3.72	1.43	0.15
Total cholesterol (mmol/L)	5.21 ± 0.81	4.95 ± 0.99	2.02	0.048
LDL-cholesterol (mmol/L)	3.29 ± 0.73	3.14 ± 0.81	1.31	0.191
HDL-cholesterol (mmol/L)	1.13 ± 0.30	1.06 ± 0.27	2.74	0.009
Triglycerides (mmol/L)	1.76 ± 1.09	1.71 ± 1.18	0.58	0.561
PANSS positive	14.14 ± 6.07	12.71 ± 5.14	1.83	0.078
PANSS negative	14.75 ± 4.86	13.79 ± 4.06	1.15	0.250
PANSS general	28.29 ± 7.19	26.79 ± 7.74	1.50	0.145
BACS Composite Z score	-1.90 ± 1.52	-1.65 ± 1.68	-1.55	0.128
BrainAGE scores	1.12 ± 2.12	0.55 ± 2.14	1.74	0.088

^a^one male participant’s endpoint weight was missing; CPZ, Chlorpromazine; Results are presented as mean ± SD; repeated-measures ANOVA by linear model (Chi-square for categorical variables) comparing baseline and endpoint. BMI, body mass index; HOMA-IR, homoeostatic model assessment for insulin resistance; HDL, high-density lipoprotein; LDL, low-density lipoprotein; PANSS, positive and negative syndrome scale.

One-way ANOVA was conducted to test the heterogeneity in ΔBMI and ΔbrainAGE among three studies. We used Shapiro-Wilk test for verifying normality (p > 0.05 for all groups). Also, homogeneity of variances was confirmed by employing Levene’s test for ΔbrainAGE(F(2, 45) = 0.01, p = 0.98) and for ΔBMI (F(2, 44) = 2.12, p = 0.13). The analysis did not show any significant difference in BMI (F F(2, 44) = 1.92, p = 0.16, η² = 0.08, 95% CI [0.00 0.23]) and brainAGE (F(2, 45) = 0.74, p = 0.48, η² = 0.03, 95% CI [0.02 0.15]) across studies. Hence, these findings demonstrate the lack of significant heterogeneity either in BMI or brainAGE among the three studies.

### Relationship between brainAGE and BMI

**Fig 1:**
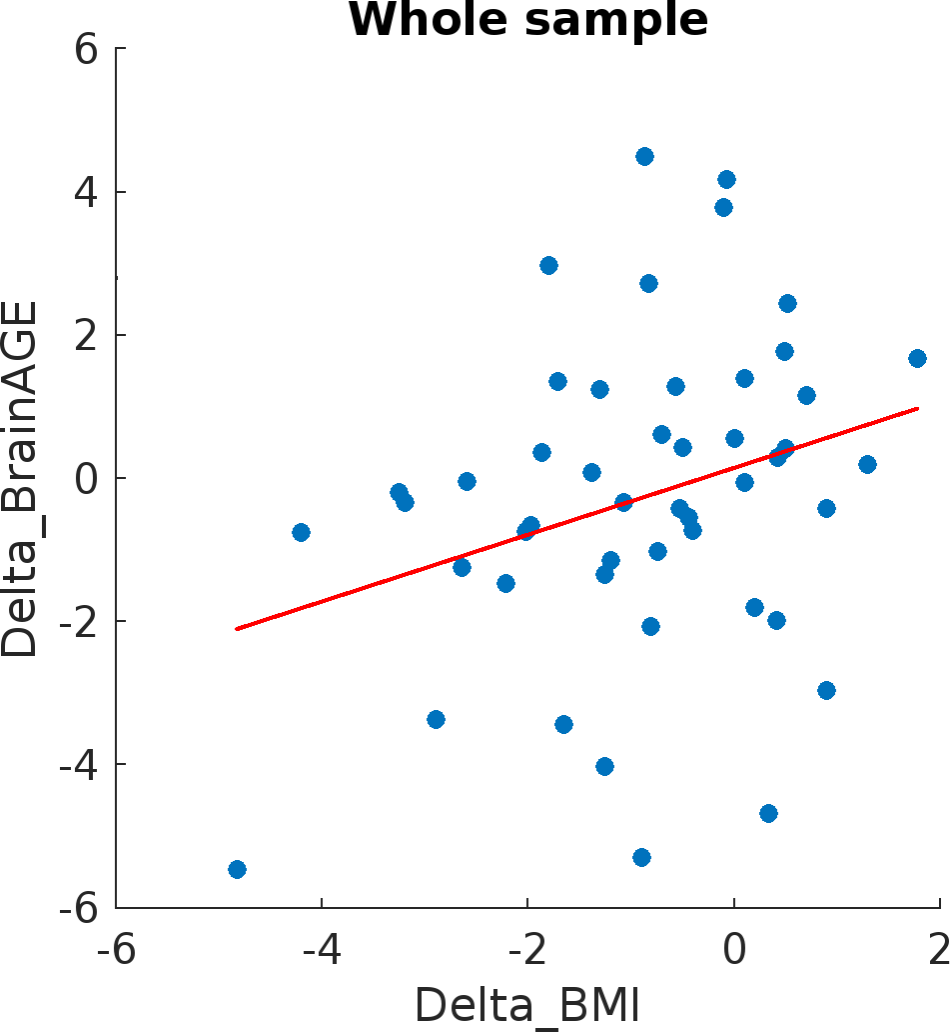
Association between ΔBMI (endpoint minus baseline denoted as Delta_BMI) and ΔbrainAGE (endpoint minus baseline labeled as Delta_brainAGE) in whole sample; brainAGE was calculated as a difference between predicted brain age and chronological age at each timepoint.

Multiple regression analysis with sex as a covariate revealed a positive association between ΔBMI and ΔbrainAGE for the whole sample (model: R^2^ = 0.14, F(2,44) = 3.69, p = 0.03; β = 0.264; Cohen f^2^ = 0.17; 95% CI [0.03 0.88]; t = 1.868; p = 0.04) (Fig 1). Moreover, adding more than one covariate did not result in any statistical significance except diabetes status as shown in the Table E in S1 Text. As an exploratory analyses, the subgroup findings are reported in the supplement Tables F and G in S1 Text and Appendix A in S1 Text.

### Relationship between brainAGE and other metabolic/clinical variables

None of the metabolic measures or psychopathology scores showed significant differences between baseline and endpoint for the whole sample. Additionally, no significant correlation was observed between the change in brainAGE and the symptom scores or other metabolic indicators (Table H in S1 Text). The analyses of correlations for the medication and placebo groups are in Tables I and J in S1 Text, respectively.

Besides, we did not see any significant association between chlorpromazine equivalents and ΔbrainAGE (r(48) = -0.07, p = 0.65). In addition, no significant correlations were observed between ΔbrainAGE and ΔΒΑCS (r(48) = -0.06, p = 0.70). The detailed subgroup results are reported in Tables I and J in S1 Text, Appendix B in S1 Text. In addition, the effects of each medication on brain structure and metabolism are given in the Appendix C in S1 Text.

## Discussion

This study focused on examining the effects of weight loss and improvements in cardiometabolic health on brainAGE. There was a notable reduction in BMI between baseline and endpoint in a whole sample of patients with SSDs who were treated for antipsychotic-induced weight gain. This significant reduction in BMI is positively correlated with the change in brainAGE. Additionally, we observed no significant associations between changes in brainAGE and any cardiometabolic or cognitive metrics.

This is the first longitudinal study to look at the relationship between reduction in BMI and brainAGE in SSDs receiving weight loss treatment. This positive correlation between ΔBMI and ΔbrainAGE is in line with a recent longitudinal study which showed that the weight loss induced by bariatric surgery contributed to reduced brainAGE compared to baseline [38]. Likewise, this same study determined that a decrease in brainAGE is related to postoperative improvements in BMI. Furthermore, post-surgery induced analyses with MRI data have reported an extensive increase in densities of grey and white matter [39]. It also resulted in an elevated resting state neural activity, suggesting an overall positive impacts of weight loss on brain status [19,40–42]. Besides, numerous neuroimaging studies have reported increase in brain volumes after aerobic exercise [43] and this was evident in schizophrenia [44]; more importantly, this may be attributable to considerable reduction in weight [45,46]. Regarding the positive association between BMI and brain age, our findings are consistent with recent neuroimaging studies on obesity [16,47], including patients with first-episode psychosis (FES) [48,49]. Specifically, a study in FEP [49] showed the negative impacts of obesity on brain and they also demonstrated that for every added point in BMI was linked to an additional month of brain ageing per year. Adding to this, a recent cross-sectional white matter volume-based study showed that overweight and obesity were linked to an estimated 10-year elevation in brain age [16]. It is proposed that obesity-linked changes in the brain’s anatomy might be due to the strain that abdominal fat places on the cardiometabolic system [50]. In summary, given the adverse impacts of obesity on brain health, our preliminary findings suggest that the weight loss may help to enhance brain health or reduce the ageing process after calorie restriction and/or weight loss [51,52].

Interestingly, this study demonstrated the present literature which suggested 4-month time frame is sufficient to assess the weight loss impacts on white matter (WM) [17,39]. This proves the fact that WM may be more vulnerable to obesity-related metabolic stress and inflammation [41]. Conversely, the metabolic improvements induced by weight loss may result in enhanced the structural integrity of WM and overall brain health. However, this short-term weight loss study may not be sufficient to capture the alterations in gray matter density (GM) and this can be owed to the modest effects of weight loss on GM. Moreover, the recent bariatric surgery-based study demonstrated that at least 12-month time period is needed to fully witness the changes caused by weight loss on GM [39]. While our study allows for witnessing the initial changes in the brain structure, we recognize that long-term impacts may not be fully captured within this limited timeframe. For example, literature shows that neuroplasticity and structural remodeling may require longer observation periods [40]. Therefore, we recommend that future studies to incorporate longer follow-up timeframes (e.g., 12–24 months) for better understanding of how metabolic interventions impacts brain anatomy in SSDs.

Recent literature has shown that the chronic alterations in satiety signaling and appetite may result in reduced inflammation and oxidative stress in the brain. Consequently, this may have positive impacts on neuroanatomy and functioning, particularly in obesity-linked conditions [53]. Furthermore, a recent meta-analysis reported that bariatric surgery induced weight loss resulted in reduced subclinical atherosclerosis and elevated positive endothelial function and hypertension [54,55]. These effects may foster angiogenesis and corresponding brain effects including enhanced synaptic connectivity, increased dendritic branching, and grey matter density [56,57]. Regardless, the lack of data on interventions aimed for treating antipsychotic-induced weight gain in psychiatric disorders has impeded our understanding about weight loss impacts on brain health.

The link between weight loss and brain age in patients with SSDs, presents significant clinical implications. Patients with these disorders often exhibit metabolic comorbid condition such as obesity. It is imperative to recognize the impacts of metabolic dysfunction which is frequently exacerbated and subsequently may also result in impaired cognition in patients with SSDs [11]. This underscores the critical need for addressing the metabolic dysfunction which may be further impaired by antipsychotics [8]. Recent studies have demonstrated that even modest weight loss over short period of time may yield significant improvements in overall brain health [58]. Specifically, weight loss may result in elevations of GM volume and improvements in cognitive performance, which continues to be an unmet need in this population [59]. Nonetheless, weight loss and maintenance in these populations are difficult due to SSDs associated with variables like antipsychotic-induced weight gain, impaired cognition, and reduced physical activity [60]. To overcome these barriers, integrated care models must implement tailored interventions including comprehensive approaches such as pharmacological, behavioral, and psychosocial support to assist weight management over a long period of time. Put together, improving metabolic health may be one way of improving brain health and cognition and our results provide initial evidence of this.

Our study findings should be interpreted with certain limitations. First, observational nature of this study impedes us from making causal inferences. Second, we acknowledge that our current dataset does not include all required variables for a thorough mediation analysis since we did not measure mediators such as inflammation and lifestyle factors. However, we encourage that future research with larger sample sizes must include mediators like inflammatory markers (such as CRP or IL-6), physical activity, diet, and sleep patterns. Third, this study included SSDs patients with comorbid type 2 diabetes/prediabetes and obesity. Thus, it was challenging to determine whether the findings were attributed solely to obesity; however, since there was no change in fasting blood glucose between baseline and endpoint, we suggest that the association was due to weight loss. For better clarity, future longitudinal studies must target enrolling patients without comorbid diabetes or other non-obese cardiovascular diseases. Fourth, although there are established differences between males and females regarding brain aging and cardiometabolic parameters, our small sample size hindered for us from analyzing by sex separately [61]. Fifth, we recognize that antipsychotics may have an effect on metabolic health and brain ageing. However, we did not observe any link between chlorpromazine equivalents and brain age which is consistent with recent literature [48,49]. To address this, perhaps future longitudinal studies can include total exposure of antipsychotics for the entire study duration. Finally, our study only accounted for factor such as medication induced weight loss. However, to determine the effects of weight loss on brain ageing accurately, future studies must incorporate influencing variables such as lifestyle modifications including improved dietary practices and increased levels of physical activity, psychosocial factors, genetics, substance use, and environmental factors.

## Conclusion

In conclusion, this study presents novel and promising initial evidence that adjunctive pharmacological interventions to improve weight outcomes among SSDs which may also decelerate the rate of brain ageing. These results suggest that substantial and ongoing weight reduction, combined with improvements in cardiometabolic disorders, can ward off the brain health complications associated with obesity.

## Supporting information

S1 Text **Table A in S1 Text**: The inclusion and exclusion criteria of three studies are listed.**Table B in S1 Text:** The Cronbach’s alpha scores for PANSS subscales for both baseline and endpoint.**Table C in S1 Text:** Characteristics of medication group at baseline and endpoint.**Table D in S1 Text:** Characteristics of placebo group at baseline and endpoint. **Table E in S1 Text:** Association between ΔbrainAGE and ΔBMI by including prediabetes/diabetes status as a covariate along with sex. **Table F in S1 Text**: Relationship between ΔbrainAGE and ΔBMI for medication and placebo group with sex as a covariate. **Table G in S1 Text**: Association between ΔbrainAGE and ΔBMI for each medicated group separately with sex as a covariate (metformin, exenatide, and topiramate). **Table H in S1 Text:** Association between ΔbrainAGE and clinical/metabolic variables in the whole study sample (medication + placebo). **Table I in S1 Text:** Association between ΔbrainAGE and clinical/metabolic variables in only medication group. **Table J in S1 Text:** Association between ΔbrainAGE and clinical/metabolic variables in placebo group. **Appendix A in S1 Text:** The association between ΔbrainAGE and ΔBMI analyzed separately for the medication and placebo group. **Appendix B in S1 Text:** Sex based stratification. **Appendix C in S1 Text:** Treatment Effects on Brain Structure and Metabolism.
(DOCX)
