# Supplementary material for: The effect of weight loss on brain age in schizophrenia spectrum disorders
Source: PLOS Ment Health. 2025 Sep 2;2(9):e0000346. doi: 10.1371/journal.pmen.0000346 (PMC12798188; doi:10.1371/journal.pmen.0000346)
Supplement: S1 Text — Table A in S1 Text: The inclusion and exclusion criteria of three studies are listed.Table B in S1 Text: The Cronbach’s alpha scores for PANSS subscales for both baseline and endpoint.Table C in S1 Text: Characteristics of medication group at baseline and endpoint.Table D in S1 Text: Characteristics of placebo group at baseline and endpoint. Table E in S1 Text: Association between ΔbrainAGE and ΔBMI by including prediabetes/diabetes status as a covariate along with sex. Table F in S1 Text: Relationship between ΔbrainAGE and ΔBMI for medication and placebo group with sex as a covariate. Table G in S1 Text: Association between ΔbrainAGE and ΔBMI for each medicated group separately with sex as a covariate (metformin, exenatide, and topiramate). Table H in S1 Text: Association between ΔbrainAGE and clinical/metabolic variables in the whole study sample (medication + placebo). Table I in S1 Text: Association between ΔbrainAGE and clinical/metabolic variables in only medication group. Table J in S1 Text: Association between ΔbrainAGE and clinical/metabolic variables in placebo group. Appendix A in S1 Text: The association between ΔbrainAGE and ΔBMI analyzed separately for the medication and placebo group. Appendix B in S1 Text: Sex based stratification. Appendix C in S1 Text: Treatment Effects on Brain Structure and Metabolism. (DOCX) [file pmen.0000346.s001.docx]

**Table A in S1 Text:** **The inclusion and exclusion criteria of three studies are listed.**

| Studies/Criteria | Exenatide | Metformin | Topiramate |
| --- | --- | --- | --- |
| Inclusion criteria | ▸ Age: 18–65 years  ▸ Diagnosis in the schizophrenia spectrum (International Classification of Diseases (ICD)-10: F20.x, F25.x)  ▸ Current and unchanged antipsychotic treatment for a minimum of 3 months  ▸ Body mass index ≥30 kg/m^2^  ▸ Glycated hemoglobin (HbA1c) <6.5% (48 mmol/mol)  ▸ Both male and female sexes (Agree to avoid pregnancy during the study) | ▸ Age: <39 years  ▸ Diagnosed with schizophrenia, schizoaffective disorder, Bipolar disorder, Major Depressive Disorder with psychotic features, or Psychotic Disorder Not Otherwise Specified (DSM V), diagnosed with prediabetes (FPG 5.6-6.9 mmol/L or 2-hour PG in the 75 g OGTT 7.8mmol/L to 11.0 mmol/l or A1c 5.7-6.4%) or Type 2 diabetes (FPG 5.6-6.9 mmol/L or 2H PG >11.1mmol/L or A1c ≥ 6.5%)  ▸ Stable early-episode outpatients or inpatients who were within 5 years of diagnosis  ▸ Body mass index ≥25 kg/m^2^  ▸ Both male and female (Agree to avoid pregnancy during the study) | ▸ Age: 17-59 years  ▸ Diagnosed with schizophrenia, or schizoaffective disorder (DSM V)  ▸ Patients must be receiving Clozapine treatment for at least 12 weeks at a dose of 350 mg/d or greater and/or have plasma CLZ levels of 300 ng/mL or greater  ▸ Clinical Global Impression Severity scale score must be 4 (4 = moderately ill) or higher and/or global assessment of functioning <50  ▸ Body mass index ≥25 kg/m^2^  ▸ Both male and female (Agree to avoid pregnancy during the study) |
| Exclusion criteria | ▸ Substance dependence (ICD-10: F1x.2 (apart from nicotine addiction F17.2))  ▸ Diabetes or HbA1c ≥6.5% (48 mmol/mol)  ▸ Contraindications to MRI (e.g.; metal implants, pacemakers, and severe claustrophobia)  ▸ Previous head trauma with a loss of consciousness for more than 5 min  ▸ Pregnancy (screened by urine human chorionic gonadotropin), lactation or no acceptance to use effective contraception during the intervention period  ▸ Severe somatic disease, including inflammatory bowel disease and nephropathy  ▸ Allergy to exenatide  ▸ Coercive measures according to the Danish Mental Health Act  ▸ Conditions that according to sponsor and/or investigators are not congruous with participation in the study  ▸ Suicidal ideations | ▸ Alcohol use disorders  ▸ patients with liver, or renal dysfunction  ▸ females of child-bearing age not on a regular contraceptive, females who are nursing, or have a positive pregnancy test will be excluded (metformin effects are unknown)  ▸ clinical or laboratory evidence of uncompensated cardiovascular, endocrine, hematological, or pulmonary disease  ▸ history of reactive hypoglycemia  ▸ HbA1c > 8.5 %, or symptomatic hyperglycemia with metabolic decompensation  ▸ prior lack of efficacy or tolerability of metformin  ▸ addition of new hypoglycemic or lipid lowering medication within 3 months of starting study  ▸ switch in antipsychotic medications within 3 months of study entry  ▸ major medical or surgical event within the preceding 3 months  ▸ acute suicidal risk | ▸ Alcohol use disorders  ▸ patients with liver, or renal dysfunction  ▸ females of child-bearing age not on a regular contraceptive, females who are nursing, or have a positive pregnancy test will be excluded  ▸ clinical or laboratory evidence of uncompensated cardiovascular, endocrine, hematological, or pulmonary disease  ▸ HbA1c > 9%, or symptomatic hyperglycemia with metabolic decompensation  ▸ prior lack of efficacy or tolerability of topiramate  ▸ addition of new hypoglycemic or lipid lowering medication within 2 months of starting study  ▸ Patients treated with hydrochlorothiazide  ▸ switch in antipsychotic medications within 3 months of study entry  ▸ major medical or surgical event within the preceding 3 months  ▸ history of renal stones  ▸ use of carbonic anhydrase inhibitor  ▸ history of glaucoma  ▸ acute suicidal risk  ▸ history of previous drug-induced encephalopathy. |

**Table B in S1 Text:** **The Cronbach’s alpha scores for PANSS subscales for both baseline and endpoint.**

| PANSS Subscale | Cronbach’s α (Baseline) | Cronbach’s α (Endpoint) |
| --- | --- | --- |
| Positive | 0.72 | 0.74 |
| Negative | 0.71 | 0.70 |
| General Psychopathology | 0.78 | 0.76 |

**Table C in S1 Text:** **Characteristics of medication group at baseline and endpoint.**

| **Variable** | **Baseline** | **Endpoint** | **t-stats** | **p-value** |
| --- | --- | --- | --- | --- |
| Sex (F:M) | 11:21 | 11:21 | - | - |
| Age (years) | 35.31 ± 9.76 | - | - | - |
| Education (years) | 13.25 ± 2.23 | - | - | - |
| Illness duration (years) | 10.87 ± 9.37 | - | - | - |
| CPZ equivalents | 543.33 ± 362.34 | - | - | - |
| Weight^a^ (kg) | 101.88 ± 22.43 | 99.63 ± 23.93 | 2.88 | **0.007** |
| BMI^a^ (kg/m2) | 34.83 ± 5.30 | 34.00 ± 5.88 | 3.07 | **0.005** |
| Waist circumference (cm) | 109.89 ± 14.46 | 106.45 ± 15.17 | 2.14 | **0.044** |
| Fasting glucose (mmol/L) | 5.56 ± 0.65 | 5.62 ± 0.69 | -0.41 | 0.686 |
| Insulin (pmol/L) | 95.91 ± 72.07 | 85.50 ± 57.41 | 0.58 | 0.567 |
| HOMA IR index | 4.09 ± 3.11 | 2.57 ± 2.06 | 2.44 | **0.021** |
| Total cholesterol (mmol/L) | 5.25 ± 0.86 | 5.03 ± 1.08 | 1.22 | 0.232 |
| LDL-cholesterol (mmol/L) | 3.35 ± 0.80 | 3.20 ± 0.89 | 0.86 | 0.398 |
| HDL-cholesterol (mmol/L) | 1.14 ± 0.34 | 1.06 ± 0.30 | 2.28 | **0.030** |
| Triglycerides (mmol/L) | 1.76 ± 1.10 | 1.79 ± 1.28 | -0.34 | 0.738 |
| PANSS positive | 13.33 ± 5.54 | 11.52 ± 4.42 | 2.11 | **0.048** |
| PANSS negative | 14.62 ± 5.14 | 13.10 ± 3.71 | 1.89 | 0.073 |
| PANSS general | 27.62 ± 7.56 | 25.57 ± 7.66 | 1.72 | 0.102 |
| BACS Composite Z score | -2.04 ± 1.64 | -1.72 ± 1.87 | -1.38 | 0.179 |

^a^: missing endpoint data for a participant; CPZ, Chlorpromazine; PANSS, positive and negative syndrome scale; BACS, Brief Assessment of Cognition in Schizophrenia; HOMA-IR, homoeostatic model assessment for insulin resistance; HDL, high-density lipoprotein; LDL, low-density lipoprotein.

**Table D in S1 Text:** **Characteristics of placebo group at baseline and endpoint.**

| **Variable** | **Baseline** | **Endpoint** | **t-stats** | **p-value** |
| --- | --- | --- | --- | --- |
| Sex (F:M) | 10:6 | 10:6 | - | - |
| Age (years) | 33.06 ± 8.23 | - | - | - |
| Education (years) | 12.17 ± 2.48 | - | - | - |
| Illness duration (years) | 12.69 ± 7.21 | - | - | - |
| CPZ equivalents | 798.33 ± 1868.93 | - | - | - |
| Weight (kg) | 109.36 ± 21.26 | 107.04 ± 21.79 | 2.54 | **0.023** |
| BMI (kg/m2) | 38.10 ± 5.35 | 37.16 ± 5.43 | 3.03 | **0.008** |
| Waist circumference (cm) | 122.00 ± 14.18 | 122.93 ± 15.01 | -0.97 | 0.364 |
| Fasting glucose (mmol/L) | 5.60 ± 0.72 | 6.44 ± 2.89 | -1.41 | 0.179 |
| Insulin (pmol/L) | 95.88 ± 34.68 | 166.38 ± 130.62 | -1.56 | 0.164 |
| HOMA IR index | 4.43 ± 2.64 | 4.39 ± 5.51 | 0.02 | 0.979 |
| Total cholesterol (mmol/L) | 5.15 ± 0.74 | 4.80 ± 0.80 | 2.47 | **0.027** |
| LDL-cholesterol (mmol/L) | 3.20 ± 0.58 | 3.02 ± 0.68 | 1.37 | 0.192 |
| HDL-cholesterol (mmol/L) | 1.13 ± 0.24 | 1.07 ± 0.23 | 1.49 | 0.158 |
| Triglycerides (mmol/L) | 1.80 ± 1.13 | 1.54 ± 0.94 | 1.52 | 0.150 |
| PANSS positive | 16.57 ± 7.39 | 16.29 ± 5.85 | 0.15 | 0.880 |
| PANSS negative | 15.14 ± 4.26 | 15.86 ± 4.67 | -0.31 | 0.768 |
| PANSS general | 30.29 ± 5.99 | 30.43 ± 7.32 | -0.081 | 0.938 |
| BACS Composite Z score | -1.60 ± 1.20 | -1.44 ± 1.22 | -1.17 | 0.260 |

CPZ, Chlorpromazine; PANSS, positive and negative syndrome scale; BACS, Brief Assessment of Cognition in Schizophrenia; HOMA-IR, homoeostatic model assessment for insulin resistance; HDL, high-density lipoprotein; LDL, low-density lipoprotein.

**Table E in S1 Text: Association between ΔbrainAGE and ΔBMI by including prediabetes/diabetes status as a covariate along with sex.**

| Study | F-stat | beta | p-value | Cohen f^2^ | 95% CI |
| --- | --- | --- | --- | --- | --- |
| Whole sample | (3,43) = 3 .04 | 0.19 | 0.04 | 0.21 | [0.01 0.80] |
| Medication | (3,27) = 4 .28 | 0.17 | 0.02 | 0.28 | [0.36 0.94] |
| Placebo | (3,12) = 1.20 | -0.10 | 0.35 | 0.14 | [-0.71 0.74] |

**Table F in S1 Text**: **Relationship between ΔbrainAGE and ΔBMI for medication and placebo group with sex as a covariate.**

| Study | F-stat | beta | p-value | Cohen f^2^ | 95% CI |
| --- | --- | --- | --- | --- | --- |
| Medication | (2,28) = 3.30 | 0.37 | 0.04 | 0.24 | [0.28 1.25] |
| Placebo | (2,13) = 0.90 | -0.10 | 0.43 | 0.11 | [-0.72 0.57] |

**Table G in S1 Text**: **Association between ΔbrainAGE and ΔBMI for each medicated group separately with sex as a covariate (metformin, exenatide, and topiramate).**

| Study | F-stat | beta | p-value | Cohen f^2^ | 95% CI |
| --- | --- | --- | --- | --- | --- |
| Topiramate | (2,9) =1.72 | 0.29 | 0.07 | 0.16 | [-0.08 1.66] |
| Exenatide | (2,6) =2.24 | 0.32 | 0.04 | 0.27 | [0.07 1.14] |
| Metformin | (2,7) =2.72 | 0.40 | 0.05 | 0.24 | [-0.01 1.79] |

**Appendix A in S1 Text: The association between ΔbrainAGE and ΔBMI analyzed separately for the medication and placebo group.**

Multiple regression analysis with sex as a covariate revealed a positive association between ΔBMI and ΔbrainAGE for the medicated group (R^2^ = 0.19, F(2,28) = 3.30, p = 0.04; β = 0.374; Cohen f2 = 0.24; 95% CI [0.28 1.25]; t = 2.141; p = 0.03), but failed to see the same association in the placebo group (R^2^ = 0.10, F(2,13) = 0.90, p = 0.43; β = -0.105; Cohen f2 = 0.11; 95% CI [-0.72 0.57]; t = -0.403; p = 0.69). Nevertheless, when we compared the correlation strength between the placebo and medication groups, we did not see any significant difference (p = 0.12). Moreover, adding more than one covariate did not result in statistical significance except diabetes status.

**Table H in S1 Text: Association between ΔbrainAGE and clinical/metabolic variables in the whole study sample (medication + placebo).**

| **Variables** | **Correlation coefficient (r)** | **p-value** |
| --- | --- | --- |
| **Demographics** | | |
| Illness duration | -0.12 | 0.40 |
| CPZ equivalents | -0.07 | 0.65 |
| **Clinical Scales** | | |
| ΔPANSS Positive | 0.15 | 0.44 |
| ΔPANSS Negative | -0.10 | 0.60 |
| ΔPANSS General | -0.19 | 0.33 |
| ΔBACS Composite | -0.06 | 0.70 |
| **Metabolic parameters** | | |
| ΔWeight^a^ | -0.21 | 0.17 |
| ΔWaist Circumference | -0.10 | 0.60 |
| ΔFasting glucose | -0.17 | 0.24 |
| ΔFasting insulin | -0.06 | 0.77 |
| ΔTotal cholesterol | 0.03 | 0.84 |
| ΔHOMA-IR | -0.03 | 0.84 |
| ΔLDL-Cholesterol | -0.05 | 0.76 |
| ΔHDL-Cholesterol | 0.03 | 0.82 |
| ΔTriglycerides | 0.19 | 0.21 |

^a^: one male participant’s endpoint weight was missing; CPZ, Chlorpromazine; PANSS, positive and negative syndrome scale; BACS, Brief Assessment of Cognition in Schizophrenia; HOMA-IR, homoeostatic model assessment for insulin resistance; HDL, high-density lipoprotein; LDL, low-density lipoprotein.

**Table I in S1 Text:** **Association between ΔbrainAGE and clinical/metabolic variables in only medication group.**

| **Variables** | **Correlation coefficient (r)** | **p-value** |
| --- | --- | --- |
| **Demographics** | | |
| Illness duration | -0.13 | 0.47 |
| CPZ equivalents | -0.05 | 0.78 |
| **Clinical Scales** | | |
| ΔPANSS Positive | 0.05 | 0.82 |
| ΔPANSS Negative | -0.07 | 0.75 |
| ΔPANSS General | -0.33 | 0.14 |
| ΔBACS Composite | -0.15 | 0.42 |
| **Metabolic parameters** | | |
| ΔWeight^a^ | -0.24 | 0.19 |
| ΔWaist Circumference | -0.04 | 0.85 |
| ΔFasting glucose | -0.15 | 0.40 |
| ΔFasting insulin | -0.13 | 0.57 |
| ΔTotal cholesterol | 0.21 | 0.25 |
| ΔHOMA-IR | -0.08 | 0.68 |
| ΔLDL-Cholesterol | 0.13 | 0.51 |
| ΔHDL-Cholesterol | -0.02 | 0.90 |
| ΔTriglycerides | 0.32 | 0.08 |

^a^: one male participant’s endpoint weight was missing; CPZ, Chlorpromazine; PANSS, positive and negative syndrome scale; BACS, Brief Assessment of Cognition in Schizophrenia; HOMA-IR, homoeostatic model assessment for insulin resistance; HDL, high-density lipoprotein; LDL, low-density lipoprotein.

**Table J in S1 Text:** **Association between ΔbrainAGE and clinical/metabolic variables in placebo group.**

| **Variables** | **Correlation coefficient (r)** | **p-value** |
| --- | --- | --- |
| **Demographics** | | |
| Illness duration | -0.08 | 0.78 |
| CPZ equivalents | 0.08 | 0.78 |
| **Clinical Scales** | | |
| ΔPANSS Positive | 0.25 | 0.55 |
| ΔPANSS Negative | -0.54 | 0.22 |
| ΔPANSS General | -0.16 | 0.73 |
| ΔBACS Composite | 0.14 | 0.62 |
| **Metabolic parameters** | | |
| ΔWeight^a^ | -0.02 | 0.94 |
| ΔWaist Circumference | 0.11 | 0.78 |
| ΔFasting glucose | -0.17 | 0.52 |
| ΔFasting insulin | 0.45 | 0.26 |
| ΔTotal cholesterol | -0.73 | 0.002 |
| ΔHOMA-IR | 0.07 | 0.79 |
| ΔLDL-Cholesterol | -0.71 | 0.003 |
| ΔHDL-Cholesterol | 0.25 | 0.38 |
| ΔTriglycerides | -0.30 | 0.28 |

CPZ, Chlorpromazine; PANSS, positive and negative syndrome scale; BACS, Brief Assessment of Cognition in Schizophrenia; HOMA-IR, homoeostatic model assessment for insulin resistance; HDL, high-density lipoprotein; LDL, low-density lipoprotein.

**Appendix B in S1 Text: Sex based stratification.**

A multiple regression analysis was conducted to examine the effects of sex, changes in BMI (ΔBMI), and their interaction term on changes in brain age (ΔbrainAGE). The overall model was not found to be significant, F(3, 43) = 2.55, p = 0.068, and it accounted for approximately 15.1% of the variance in ΔbrainAGE (R² = 0.151). These findings suggest that changes in BMI are associated with changes in brain age; however, this relationship does not significantly differ between males and females. It is strongly recommended that the future research with larger sample sizes be undertaken to confirm these outcomes.

**Appendix C in S1 Text: Treatment Effects on Brain Structure and Metabolism.**

Metformin, commonly prescribed medication for type 2 diabetes is known to elevate insulin sensitivity and decreases hepatic glucose production and eventually, this aids with metabolic regulation (Foretz et al., 2023). Furthermore, recent studies have shown that metformin may offer neuroprotective benefits including improved cognitive function and reduced neuroinflammation by activating AMPK and enhancing mitochondrial function (Ng et al., 2014; Kulkarni et al., 2020). Likewise, GLP-1 receptor agonist (exenatide) is also recommended in managing diabetes. It supports the mechanism of glucose-dependent insulin secretion and inhibiting glucagon release and subsequently, aiding in weight loss (Drucker & Nauck, 2006). Importantly, exenatide also demonstrated the neuroprotective effects such as mitigating neuroinflammation and improving cognitive function through GLP-1 receptor signaling (Gejl et al., 2016; Athauda et al., 2017). Whereas, topiramate is an anticonvulsant which works via modulating neuronal excitability. It is known for enhancing GABAergic activity and inhibiting glutamate receptors which makes it more impactful for treatments such as epilepsy and migraine (Shank et al., 2000). Importantly, it also exhibits weight loss properties through elevating feelings of fullness and suppressing appetite (Bray et al., 2023). This is the first exploration study that made use of these medications for treating weight gain induced by antipsychotics in SSDs. Due to the limited literature available on the impacts of these medications on brain anatomy, we consider this study to serve as a foundation for future studies in this field of research. In summary, our preliminary findings have highlighted the benefits of using these medications for targeting both metabolic and neurological pathways in SSDs.

### **References**

1. Foretz, M., Guigas, B., & Viollet, B. (2023). Metformin: Update on mechanisms of action and repurposing potential. *Nature Reviews Endocrinology, 15*(10), 569-589.
2. Ng, T. P., Feng, L., Yap, K. B., Lee, T. S., Tan, C. H., & Winblad, B. (2014). Long-term metformin usage and cognitive function among older adults with diabetes. *Journal of Alzheimer's Disease, 41*(1), 61-68.
3. Kulkarni, A. S., Gubbi, S., & Barzilai, N. (2020). Benefits of metformin in attenuating the hallmarks of aging. *Cell Metabolism, 32*(1), 15-30.
4. Drucker, D. J., & Nauck, M. A. (2006). The incretin system: glucagon-like peptide-1 receptor agonists and dipeptidyl peptidase-4 inhibitors in type 2 diabetes. *The Lancet, 368*(9548), 1696-1705.
5. Gejl, M., Gjedde, A., Egefjord, L., Møller, A., Hansen, S. B., Vang, K., ... & Brock, B. (2016). In Alzheimer’s disease, six-month treatment with GLP-1 analog prevents decline of brain glucose metabolism: randomized, placebo-controlled, double-blind clinical trial. *Frontiers in Aging Neuroscience, 8*, 108.
6. Athauda, D., Maclagan, K., Skene, S. S., Bajwa-Joseph, M., Letchford, D., Chowdhury, K., & Foltynie, T. (2017). Exenatide once weekly versus placebo in Parkinson’s disease: a randomised, double-blind, placebo-controlled trial. *The Lancet, 390*(10103), 1664-1675.
7. Shank, R. P., Gardocki, J. F., Streeter, A. J., & Maryanoff, B. E. (2000). An overview of the preclinical aspects of topiramate: pharmacology, pharmacokinetics, and mechanism of action. *Epilepsia, 41*(s1), S3-S9.
8. Bray, G. A., Hollander, P., Klein, S., Kushner, R., Levy, B., Fitchet, M., & Perry, B. H. (2003). A 6-month randomized, placebo-controlled, dose-ranging trial of topiramate for weight loss in obesity. *Obesity Research, 11*(6), 722-733.
